# Transcultural adaptation to Brazilian Portuguese and reliability of the effort-reward imbalance in household and family work

**DOI:** 10.1590/S1518-8787.2016050006138

**Published:** 2016-06-17

**Authors:** Ilmeire Ramos Rosembach de Vasconcellos, Rosane Härter Griep, Luciana Portela, Márcia Guimarães de Mello Alves, Lúcia Rotenberg

**Affiliations:** I Programa de Pós-Graduação em Saúde Pública. Escola Nacional de Saúde Pública. Fundação Oswaldo Cruz. Rio de Janeiro, RJ, Brasil; II Laboratório de Educação em Ambientes e Saúde. Instituto Oswaldo Cruz. Fundação Oswaldo Cruz. Rio de Janeiro, RJ, Brasil; III Instituto de Saúde Coletiva. Universidade Federal Fluminense. Niterói, RJ, Brasil

**Keywords:** Homemaker Services, Evaluation, methods, Questionnaires, Translations, Reproducibility of Results, Validation Studies

## Abstract

**OBJECTIVE:**

To describe the steps in the transcultural adaptation of the scale in the Effort-reward imbalance model to household and family work to the Brazilian context.

**METHODS:**

We performed the translation, back-translation, and initial psychometric evaluation of the questionnaire that comprised three dimensions: (i) effort (eight items, emphasizing quantitative workload), (ii) reward (11 items that seek to capture the intrinsic value of family and household work, societal esteem, recognition from the spouse/partner, and affection from the children), and (iii) overcommitment (four items related to intrinsic effort). The scale was included in a sectional study conducted with 1,045 nursing workers. A subsample of 222 subjects answered the questionnaire for a second time, seven to 15 days thereafter. The data were collected between October 2012 and May 2013. The internal consistency of the scale was evaluated using Cronbach’s alpha and test-retest reliability analysis, square weighted kappa, prevalence and bias adjusted Kappa, and intraclass correlation coefficient.

**RESULTS:**

Prevalence and bias-adjusted Kappa (k_a_) of the scale dimensions ranged from 0.80-0.83 for overcommitment, 0.78-0.90 for effort, and 0.76-0.93 for reward. In most dimensions, the values of minimum and maximum scores, average, standard deviation, and Cronbach’s alpha were similar in test and retest scores. Only on societal esteem subdimension (reward) was there little variation in standard deviation (test score of 2.24 and retest score of 3.36) and in Cronbach’s alpha coefficient (test score of 0.38 and retest score of 0.59).

**CONCLUSIONS:**

The Brazilian version of the scale was found to have proper reliability indices regarding time stability, which suggests adapting it to be used in population with characteristics that are similar to the one in this study.

## RESUMO

**OBJETIVO:**

Descrever as etapas da adaptação transcultural da escala do *Effort-reward imbalance model to household and family work* para o contexto brasileiro.

**MÉTODOS:**

Efetuou-se a tradução, retrotradução e avaliação psicométrica inicial do instrumento composto por três dimensões: (i) esforço (oito itens, enfatizando a carga quantitativa de trabalho), (ii) recompensa (11 itens que buscam captar o valor intrínseco da família e do trabalho doméstico, a estima social, o reconhecimento do cônjuge ou companheiro e a afeição dos filhos) e (iii) o excesso de comprometimento (quatro itens relacionados ao esforço intrínseco). A escala foi incluída em um estudo seccional aplicado em 1.045 trabalhadoras de enfermagem. Uma subamostra de 222 participantes respondeu ao questionário pela segunda vez, com intervalo de sete a 15 dias. Os dados foram coletados entre outubro de 2012 e maio de 2013. A consistência interna da escala foi avaliada pelo coeficiente de alpha de Cronbach e a confiabilidade teste-reteste, pelo índice kappa ponderado quadrático, pelo kappa ajustado pela prevalência e pelo coeficiente de correlação intraclasse.

**RESULTADOS:**

A confiabilidade ajustada pela prevalência (k_a_) das dimensões da escala variou de 0,80-0,83 para o excesso de comprometimento, 0,78-0,90 para o esforço e 0,76-0,93 para a recompensa. Na maioria das dimensões, os valores do escore mínimo e máximo, média, desvio-padrão e alpha de Cronbach no teste e no reteste foram semelhantes. Somente na subdimensão estima social (recompensa) houve pequena variação no desvio padrão (2,24 no teste e 3,36 no reteste) e no coeficiente de alpha de Cronbach (0,38 no teste e 0,59 no reteste).

**CONCLUSÕES:**

A versão brasileira da escala apresentou índices adequados de fidedignidade quanto à estabilidade temporal, o que sugere a adequação da escala para uso em populações com características semelhantes à do estudo.

## INTRODUCTION

Several studies point towards negative health impacts from household and family work. A Brazilian study with 2,057 women from Feira de Santana, BA, Northeastern Brazil, found significant association between household and family work overload and the most common disorders, which are characterized by symptoms such as fatigue, memory lapses, insomnia, irritability, difficulty concentrating, headaches, and psychosomatic symptoms^8^. Another line of studies discusses household and family work demands in combination with stress at professional work, and problems were identified regarding depression^5^, common mental disorders and difficulty recovering after professional work^12^, and arterial blood pressure changes^2^ ^,^ ^9^. Despite the evidence suggesting household and family work may be a source of diseases and physical exhaustion; up until recently no specific tools to evaluate psychosocial stress from household and family work existed.

In 2012, Sperlich et al.^17^ proposed an adaptation of the effort-reward imbalance model (ERI) to the household environment^15^. Such tool is recognized in the occupational health field as adequate to evaluate stress from professional work. The ERI model^15^ considers stress as the result from an imbalance between effort that is made and rewards that are received due to work. Thus, the higher the effort (a worker’s duties), the smaller the reward (support and respect from colleagues, proper wages, possibility of promotions, tenure, and social status), causing a higher imbalance that could generate frustration and feelings of injustice^15^. A third dimensions is part of the model – overcommitment to work –, which is an internal component of effort that is related to a worker’s personality and to the way they deal with their work requirements. This dimension supposedly acts by changing the effects from the negative consequences from the imbalance between effort and reward at work^15^. Several studies found an association between ERI and the different health outcomes, such as arterial hypertension^20^, low quality of life^18^, and physical and psychic morbidity symptoms^13^.

According to Sperlich et al.^17^, household and family work, as well as professional work, has a social identity and can be equally strenuous and gratifying, thus implying costs and gains. However, its demands can be less obvious, once the basic household chores are considered to be “natural” responsibilities of women. The rewards are generally emotional in nature, such as the social acknowledgment of the role mothers and wives perform, and the social affection from their children and husbands. Thus, generalizing reward aspects in the professional environment (related to issues regarding financial matters, careers, esteem, gratification, and job security) to unpaid work is not possible. In this context, the authors adapted the ERI model to household and family work performed by women^17^.

Sperlich et al.^17^ evaluate that, as posited by the ERI model, stress in household and family work is tied to the dynamics between effort and reward. Thus, when there is an imbalance between the high effort performed at household and family work and the low reward received from children or partners, emotions such as anger and frustration could arise as a result from the feeling of having been treated unfairly, which causes both stress and sickening. In this perspective, the Effort-Reward Imbalance in Household and Family Work (herein referred to as “domestic ERI”) focuses on the peculiarities of household and family work, which is still predominantly performed by women^4^. Effort is measured by the workload in activities such as cooking, washing and ironing, tidying up and cleaning the house, and organizing tasks related to family and child care. Reward is measured by considering the intrinsic value of family and household work, societal esteem from work as mothers and wives, recognition from spouses or partners, and affection from children. Still in a way that is similar to the original ERI model, we included the “overcommitment to household and family work” dimension to the new scale. This dimension refers to the excessively motivational character related to work. People with that characteristic have increased risk of experiencing imbalance between costs and gains, as they tend to be too invested at work. Thus, the high effort made rarely finds proper reward^19^.

Domestic ERI comprises 23 items based on women’s demands regarding their daily domestic environments and are divided in three dimensions: (i) effort, which is measured using eight items concerning work overload and household chores; (ii) reward, which comprises 11 items divided in four subdimensions (intrinsic value of family and household work, societal esteem, recognition from spouses or partners, and affection from children); and (iii) overcommitment, which is evaluated according to four items regarding the component of personal nature (intrinsic effort), which evaluates a woman’s ability to have herself removed from household and family duties. This scale was developed and validated in a study in Germany with 3,129 women with children younger than 18 years old^17^. Its results pointed towards a factorial structure compatible with the theoretical imbalance model between effort and reward. Besides that, family ERI was found to be associated with milder psychic disorders (anxiety and depression), with worse self-reported health statuses, and to higher arterial blood pressure levels^17^.

This article aimed to describe the steps in the transcultural adaptation of domestic ERI scale to the Brazilian context.

## METHODS

**Figure 1:**
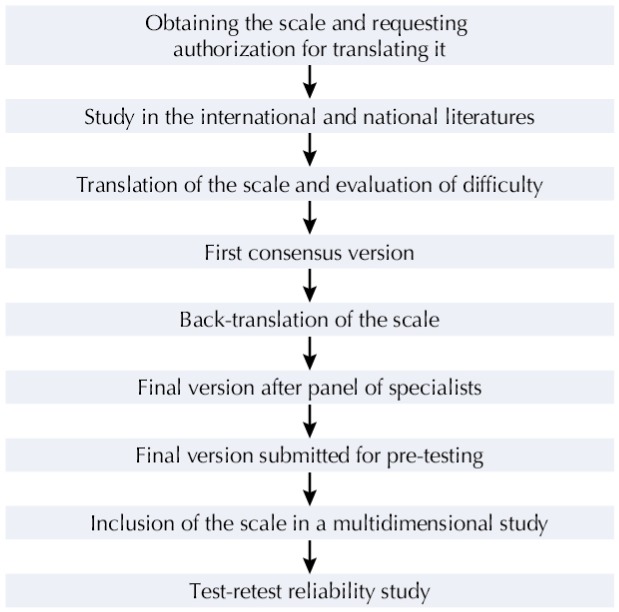
Scheme of steps in the transcultural adaptation of the Effort-reward imbalance model to household and family work scale.

The process of adapting the domestic ERI scale to the Brazilian cultures, shown in Figure 1, has followed the recommendations from Herdman et al.^6^ and Reichenheim and Moraes^10^.

The translation was performed by three independent native Brazilian Portuguese-speaking translators, asked to give grades indicating the difficulty level to translate each item in the scale, on a specific form. These scores ranged from zero (no difficulty) to 10 (maximum difficulty). According to these instructions, the priority was to translate the meanings of terms (semantic equivalence) rather than to just translate excerpts literally.

In compliance with the authors of the original scale on household and family work^17^, recent recommendations were followed regarding the formats of answer categories to the items in the ERI scale^14^. A Likert scale was adopted (completely disagree; partially disagree; partially agree; completely agree) (operational equivalence).

The first consensus version for the three translations of the scale was obtained by a panel of specialists (two epidemiologists and two workers’ health care researchers) that were experienced in the use of scales and in the adaptation of questionnaires to Brazilian Portuguese. This version was pre-tested with eight women who had been asked to review how clearly they could understand the items.

A new consensus version containing changes suggested in the tests was the submitted to two native English-speaking translators who independently retranslated the Brazilian version of the scale (first version of the scale) back to English. They had no access to the original English version of the scale.

After that, the specialists compared the original English version, its translation to Portuguese, and the back-translation, and achieved the final translated version of the scale. Doubts on the most adequate translations for the terms were discussed with one of the authors of the original scale, Stephanie Sperlich. This version, which was obtained from previous steps, was submitted to two pre-testing rounds, to evaluate for a second time the clarity and appropriateness of the terms in the Brazilian culture.

The final version of the scale was inserted into a multidimensional questionnaire, which was applied to a group of female workers from a large general hospital in Rio de Janeiro. The data were collected from October 2012 to May 2013, on the hospital’s different work shifts and on the seven days of the week. The questionnaire was filled by the subjects with help from trained interviewers. Among the 1,332 eligible professionals, 1,224 workers (91.9%) took part in the study. Losses regarded to refusals (81) and subjects not found (27), totaling 108 workers (8.8%). Only women were included in the presented analyses (n = 1,045).

A convenience subsample (n = 222) took part in the test-retest reliability study to test for time stability of the questionnaire. The nursing workers (nurses, nursing technicians, and nursing auxiliaries) were invited to fill out the same questionnaire again after seven to 15 days.

After being filled out, the questionnaires were revised by a trained research assistant. The answers obtained in the sectional study and in the test-retest reliability analysis were double typed into a database (EpiInfo software, version 3.5.4), and had their inconsistencies rectified. The data were analyzed in softwares Statistical Package for Social Science for Windows (SPSS, version 20) and Computer Programs for Epidemiologists for Windows (WinPepi, version 11.39).

The domestic ERI scores were calculated by adding the points in each alternative in dimensions effort, reward, and overcommitment, whose points were distributed as follows: (1) completely disagree, (2) partially disagree, (3) partially agree, and (4) completely agree. The following items from reward dimension had their scores inverted: “from my child/children I usually feel the appreciation and affection that I would wish for”, “I receive a great deal in return from my child/children for my efforts at home”, “I feel that overall, household and family work are worth the effort”, “The work I do for my family provides a deeper meaning to my life”, “I usually obtain an appropriate level of recognition and appreciation from my partner for my work at home”, and “my partner often thanks me for my work at home”. This inversion was necessary so the same pattern in the answers to the remaining questions, whose scores increase as the negative evaluation of each item increases, could be followed. The imbalance between effort and reward was calculated according the following equation: Domestic ERI = *e/(r x c)*, where *e* is the sum of the scores of effort items, *r* is the sum of reward scores and *c* is the 0.73 correction factor, which derives from the division between the number of items regarding effort and reward (8/11). Values above 1 indicate imbalance in the relationships between effort and reward, which means that the effort made outweighs the reward received in regards to household and family work. The commitment to household chores and family duties was evaluated by summing the scores in each item. Equal results of the ones above 12 indicate overcommitment^17^.

The internal consistency of the items that compose each dimension of the scale was evaluated using Cronbach’s alpha. Time stability (test-retest reliability analysis) was evaluated by the square weighted kappa index with its respective 95% confidence intervals (95%CI) and the prevalence and bias-adjusted Kappa (PABAK)^3^, calculated in the Winpepi software (version 11.39). Test-retest reliability of dimensions was evaluated using the intraclass correlation coefficient (ICCC) with its 95%CI (SPSS, version 20). Byrt’s criteria^3^ were adopted for interpreting the reliability results in the study, as follows: weak (0 to 0.20), mild (0.21 to 0.40), reasonable (0.41 to 0.60), good (0.61 to 0.80), very good (0.81 and 0.92), and excellent (0.93 to 1.00) (measurement equivalence).

The research was approved by Oswald Cruz Foundation’s Human Research Ethics Committee – Oswaldo Cruz Institute (CEP Fiocruz-IOC: 635/11). All subjects signed consent forms.

## RESULTS

The marks given by the translators regarding the difficulty level of their translations ranged from zero to three. During the translation process, the longer headers caused more doubts, which were properly resolved while the consensus version was drafted. This version was sent for back-translation, which produced two English versions. After that, the original version, the back translations, and the final translation were compared by the panel of specialists. The professionals in this panel considered the difficulties pointed out by the translators in the first step and by the back translators, and attempted to achieve a pre-test version. This process also generated instructions to be observed in the following pre-test stage.

The professionals overseeing the subjects filling out the questionnaires were requested to solve the respondents’ doubts regarding the questions in both pre-test stages. The remaining doubts were solved by comparing the original German, the English, and the Portuguese versions. The original author of the scale, Stephanie Sperlich, was also inquired.

During these stages, four items in the scale caused doubts, and they were discussed with one of the scale’s authors. The items were the following:

1) In the case of item “I easily run into time pressures in my household and family work”, the doubt regarded the best adaptation for the expression “run into time pressures” based on what the original version intended to convey. The issue was: was the person being pressed for time due to excess duties (i.e., “being subject to time pressure”) or feeling pressed because of their own private feelings? Reward dimension can be observed to have an item that concerns to time pressure (There is often great time pressure because of the many household and family duties). Thus, for the first item to be able to capture the intrinsic dimension regarding overcommitment, the final translation, in Portuguese, “I am easily subjected to time pressure during household and family work”.

2) Item “Nowadays, a person is regarded disapprovingly if he/she is ‘only’ involved in household and family work*”* brought the word ‘only’ between quotes. We understand that emphasis was placed on the word ‘only’, as if the idea of someone working at home performing household chores only, out of the job market, could not be conceived. In the Brazilian reality, depending on a person’s social class, that is a frequent situation. We talked to one of the authors of the original scale about the intended use of quotes, and she suggested they be removed.

3) Item *“*In my interactions with other people, I often have the experience that the roles of housewife and mother are poorly recognized and appreciated*”* caused doubts regarding what was sought to be measured in the seemingly vague Portuguese phrase “*nas minhas interações com outras pessoas*”, a literal translation of *“*in my interactions with other people*”*. Thus, we chose to maintain the meaning, although if it was not literal, and the phrase was replaced by “*Quando me relaciono com outras pessoas*” (“When I interact with other people”).

4) The doubt caused by item “Often my partner does not notice my work in the household and for the family” regarded the phrase “does not notice”, which, in the translation that was proposed by the translators, became “*não nota ou não vê*” (does not notice or see it). After the pre-tests and the discussion with one of the authors of the original scale, the translation of this item was “*Muitas vezes meu parceiro não enxerga o meu trabalho doméstico e familiar*” (“My partner oftentimes does not see the work I do around the house”) as it was easier for respondents to understand.

The original version and the final version of the scale, which was obtained after the adaptation process, are shown in Table 1.

**Table 1:** Questionnaire for measuring effort-reward imbalances in household and family work, in its original English version* and in its final Portuguese version.

Domestic ERI	Original English version	Final Portuguese version
Overcommitment	1. From the moment I wake up in the morning, I often begin to worry about household and family work that needs to be completed.	*1. Desde que eu acordo eu começo a me preocupar com o trabalho doméstico e familiar que preciso fazer.*
	2. I constantly think about my responsibilities at home, and I’m still preoccupied with them in the evening.	*2. Eu penso constantemente nas minhas responsabilidades domésticas e continuo preocupada com elas à noite.*
	3. I easily run into time pressures in my household and family work.	*3. Eu facilmente estou sujeita à pressão do tempo no trabalho doméstico e familiar.*
	4. If I postpone something that I really should have finished today, I have trouble sleeping at night.	*4. Eu tenho dificuldade para dormir se eu adiar algo que deveria ter terminado naquele dia.*
Effort	1. Frequently there is great time pressure due to the many tasks in household and for my family.	*1. Frequentemente existe uma grande pressão de tempo por conta das muitas tarefas domésticas e familiares.*
	2. I am frequently interrupted and disturbed in my activities in the household and for my family.	*2. Eu sou frequentemente interrompida e incomodada nas minhas atividades domésticas e familiares.*
	3. Often I feel as never being off duty.	*3. Muitas vezes eu sinto como se nunca tivesse folga.*
	4. I would need more hours in the day in order to accomplish all my household and family work.	*4. Eu precisaria de mais horas no dia para concluir todo o meu trabalho doméstico e familiar.*
	5. Over the last years, my household and family work have become more extensive.	*5. Nos últimos anos, meu trabalho doméstico e familiar tem aumentado.*
	6. In household and family work, I often have the feeling of having to accomplish ‘a thousand things’ all at the same time.	*6. Muitas vezes eu tenho a sensação de ter que fazer “mil coisas” ao mesmo tempo no trabalho doméstico e familiar.*
	7. I often feel overwhelmed by the large number of household and family responsibilities.	*7. Muitas vezes eu me sinto sobrecarregada pelo grande número de responsabilidades domésticas e familiares.*
	8. I hardly get a moment’s rest during the day because of the many demands placed on me by the household and my family.	*8. É difícil eu ter um momento de descanso durante o dia, por conta das muitas demandas domésticas e familiares.*
Reward	Intrinsic value 1. I feel that overall, household and family work are worth the effort.	*Valor intrínseco 1. Em geral, eu sinto que o esforço no trabalho doméstico e familiar vale a pena.*
	2. I often question the meaning of household and family work, since I have to start all over again every day.	*2. Eu frequentemente questiono o sentido do trabalho doméstico e familiar, já que tenho que começar tudo de novo a cada dia.*
	3. The work I do for my family provides a deeper meaning to my life.	*3. O trabalho que eu faço para a minha família dá um significado mais profundo à minha vida.*
	Societal esteem 4. In my interactions with other people, I often have the experience that the roles of housewife and mother are poorly recognized and appreciated.	*Estima social 4. Quando me relaciono com outras pessoas, muitas vezes sinto que os papéis de dona de casa e de mãe são pouco reconhecidos e valorizados.*
	5. Nowadays, a person is regarded disapprovingly if he/she is ‘only’ involved in household and family work.	*5. Hoje em dia, uma pessoa é vista com desaprovação se estiver envolvida apenas com o trabalho doméstico e familiar.*
	6. The fact that household and family work are unpaid seems unjust to me.	*6. Eu acho injusto o trabalho doméstico e familiar não serem remunerados.*
	Recognition from the partner 7. I usually obtain an appropriate level of recognition and appreciation from my partner for my work at home.	*Reconhecimento do parceiro 7. Meu parceiro dá o devido reconhecimento e valor pelo meu trabalho em casa.*
	8. Often my partner does not notice my work in the household and for the family.	*8. Muitas vezes meu parceiro não enxerga o meu trabalho doméstico e familiar.*
	9. My partner often thanks me for my work at home.	*9. Meu parceiro geralmente agradece pelo meu trabalho em casa.*
	Affection from the children 10. From my child/children I usually feel the appreciation and affection that I would wish for.	*Reconhecimento dos filhos 10. Meus filhos me dão o valor e o afeto que eu gostaria de receber.*
	11. I receive a great deal in return from my children/child for my efforts at home.	*11. Meus filhos reconhecem o meu esforço em casa.*

ERI: effort-reward imbalance

* Granted by Stefanie Sperlich.

Although the subsample of nursing workers has been obtained by convenience in the test-retest reliability analysis, their sociodemographic characteristics were very similar to the ones of the population in the sectional study (Table 2). The average age of the respondents was 45 years; more than half of them had attended college; and a third of them reported that their families earned income of up to two times the minimum monthly wage. More than half of them were married, and around one fourth of the subjects reported having children under six years of age. Regarding their occupational characteristics, most subjects were observed to work as nursing auxiliaries; they reported spending 24.5 hours weekly with household and family duties in average, and spending 33.5 hours weekly with their professional duties in average. However, a significant difference was observed in the range of hours spent with domestic and professional duties in the subsample. The time spent with household chores, as reported in the sectional study, was 20 to 106 hours; in the test-retest reliability analysis, it ranged between 0.5 and 102 hours. The time spent with professional duties, as reported in the sectional study, was from four to 105 hours; in the test-retest reliability analysis, it ranged between six and 72 hours.

**Table 2:** Sociodemographic and occupational characteristics of the sectional study subjects (n = 1,045) and of the test-retest reliability analysis of domestic ERI (n = 222). Rio de Janeiro, RJ, Southeastern Brazil, 2013.

Sociodemographic and occupational characteristics	Sectional study		Test-retest reliability study	
	
	n	%	n	%
Age (in years)				
Mean (SD)	44.3 (11.2)	-	45.3 (11.7)	-
Variation	25-69	-	26-69	-
Education level				
Elementary education	29	2.8	7	3.2
High school education	358	34.3	90	40.5
College education	658	63.0	125	56.3
*Per capita* income in minimum monthly wages*				
Up to two minimum monthly wages	338	32.3	75	33.8
Between two and four minimum monthly wages	456	43.6	93	41.9
Over four minimum monthly wages	237	22.7	52	23.4
No information	14	1.4	2	0.9
Marital status				
Married or common-law marriage	582	55.7	121	54.5
Separated or divorced	162	15.5	25	11.2
Widow	35	3.3	11	5.0
Single	266	25.5	65	29.3
Children under the age of 6 living with you				
Yes	235	22.5	48	21.6
No	806	77.1	174	78.4
No information	4	0.4	-	-
Job				
Nurse	360	34.4	78	35.1
Technician	152	14.5	32	14.4
Auxiliary	533	51.0	112	50.5
Time dedicated to domestic duties over the last week (in hours)				
Mean (SD)	22.7 (17.3)	-	24.5 (18.5)	-
Variation	20-106	-	0.5-102	-
No information	43	-	11	-
Time dedicated to professional duties over the last week (in hours)				
Mean (SD)	35.0 (15.9)	-	33.5 (17.7)	-
Variation	4-105	-	6-72	-
No information	28	-	5	-

ERI: effort-reward imbalance

* Minimum monthly wage in December 2012 = R$678,00.

The time stability of each item in the dimensions that compose household and family ERI questionnaire is shown in Table 3. The prevalence and bias-adjusted Kappa (PABAK) values ranged from 0.80 to 0.83 (good to very good) for items overcommitment, from 0.78 to 0.90 (good to very good) for items related to effort, and from 0.76 to 0.93 (good to excellent) for the reward items. Generally speaking, time stability values increased after being adjusted for prevalence for most items.

**Table 3:** Test-retest reliability analysis of the items in the effort-reward imbalance model to household and family work questionnaire. (N = 222)

Domestic ERI	Items	Square weighted kappa	95%CI	PABAK
Overcommitment	1. From the moment I wake up in the morning, I often begin to worry about household and family work that needs to be completed.	0.65	0.56–0.74	0.82
	2. I constantly think about my responsibilities at home, and I’m still preoccupied with them in the evening.	0.65	0.56–0.74	0.80
	3. I easily run into time pressures in my household and family work.	0.71	0.63–0.78	0.83
	4. If I postpone something that I really should have finished today, I have trouble sleeping at night.	0.68	0.59–0.77	0.81
Effort	1. Frequently there is great time pressure due to the many tasks in household and for my family.	0.72	0.65–0.80	0.84
	2. I am frequently interrupted and disturbed in my activities in the household and for my family.	0.68	0.60–0.76	0.84
	3. Often I feel as never being off duty.	0.66	0.56–0.75	0.80
	4. I would need more hours in the day in order to accomplish all my household and family work.	0.64	0.55–0.74	0.78
	5. Over the last years, my household and family work have become more extensive.	0.80	0.75–0.86	0.88
	6. In household and family work, I often have the feeling of having to accomplish ‘a thousand things’ all at the same time.	0.73	0.66–0.80	0.84
	7. I often feel overwhelmed by the large number of household and family responsibilities.	0.77	0.71–0.84	0.90
	8. I hardly get a moment’s rest during the day because of the many demands placed on me by the household and my family	0.74	0.66–0.82	0.84
Reward	Intrinsic value 1. I feel that overall, household and family work are worth the effort	0.54	0.43–0.66	0.84
	2. I often question the meaning of household and family work, since I have to start all over again every day.	0.53	0.43–0.64	0.76
	3. The work I do for my family provides a deeper meaning to my life.	0.58	0.47–0.69	0.85
	Societal esteem 4. In my interactions with other people, I often have the experience that the roles of housewife and mother are poorly recognized and appreciated.	0.53	0.42–0.63	0.76
	5. Nowadays, a person is regarded disapprovingly if he/she is ‘only’ involved in household and family work.	0.55	0.44–0.65	0.76
	6. The fact that household and family work are unpaid seems unjust to me.	0.71	0.62–0.79	0.83
	Recognition from the partner 7. I usually obtain an appropriate level of recognition and appreciation from my partner for my work at home.	0.81	0.73–0.90	0.93
	8. Often my partner does not notice my work in the household and for the family.	0.67	0.55–0.79	0.83
	9. My partner often thanks me for my work at home.	0.62	0.47–0.77	0.83
	Affection from the children 10. From my child/children I usually feel the appreciation and affection that I would wish for.	0.39	0.23–0.56	0.81
	11. I receive a great deal in return from my children/child for my efforts at home.	0.65	0.52–0.78	0.88

ERI: effort-reward imbalance; PABAK: prevalence-adjusted and bias adjusted kappa

The four items that were found to be the most difficult to translate, which were included in the probing stage, had the following values for their kappa indices: “I easily run into time pressures in my household and family work” (kappa = 0.83; very good); “In my interactions with other people, I often have the experience that the roles of housewife and mother are poorly recognized and appreciated” (kappa = 0.76; good); “Nowadays, a person is regarded disapprovingly if he/she is ‘only’ involved in household and family work” (kappa = 0.76; good); and “Often my partner does not notice my work in the household and for the family” (kappa = 0.83; very good).

The descriptive statistics and the reliability of dimensions and sub-dimensions proposed by the original version of the questionnaire are shown in Table 4. In most dimensions, the values of minimum and maximum scores, average, standard deviation, and Cronbach’s alpha were similar in test and retest analyses. On societal esteem subdimension (reward), we observed little variation in standard deviation (test score of 2.24 and retest score of 3.36) and in Cronbach’s alpha coefficient (test score of 0.38 and retest score of 0.59). The values of the intraclass correlation coefficients ranged from 0.89 to 0.93 (very good to excellent) for dimensions overcommitment, effort, and global reward; in the reward sub-dimensions, they ranged from 0.78 (good) for “intrinsic value” to 0.88 (very good) for “recognition from the partner”. Reward dimension comprises 11 items; however, five of them were not applied to the unmarried or childless subjects. In this case, some subjects in the study only answered six questions, which justifies the minimum score of 6 for test and retest evaluations.

**Table 4:** Mean, standard deviation, and Cronbach’s alpha coefficient of the scores in the dimensions of the effort-reward imbalance scale in household and family work. Test-retest reliability study Rio de Janeiro, RJ, Southeastern Brazil, 2013. (N = 222)

Dimensions	no. of items	Test				Retest				ICCC	95%CI
		
Domestic ERI		Score (Min and Max)	Average Score	SD	Cronbach’s alpha	Score (Min and Max)	Average Score	SD	Cronbach’s alpha		
Overcommitment											
	4	4-16	9.7	3.5	0.76	4-16	10.05	3.5	0.79	0.89	0.86–0.92
Effort	8	8-32	20.0	7.4	0.93	8-32	20.5	7.6	0.93	0.93	0.91–0.95
Global reward	11	6-39	24.4	5.7	0.76	6-38	24.8	5.6	0.76	0.93	0.91–0.94
Reward dimensions											
Intrinsic value	3	3-12	6.38	1.87	0.32	3-12	6.38	1.69	0.39	0.78	0.72–0.87
Societal esteem	3	3-12	8.10	2.24	0.38	3-12	8.60	3.36	0.59	0.79	0.73–0.84
Recognition from the partner	3	3-12	6.29	2.65	0.82	3-12	6.53	2.42	0.76	0.88	0.83–0.92
Affection from the children	2	2-7	3.37	1.32	0.55	2-7	3.62	1.58	0.61	0.87	0.84–0.90

ERI: effort-reward imbalance; ICCC: intraclass correlation coefficient with 95%CI

Evaluating the internal consistency of each subdimension in the scale in case any items were removed was shown to decrease the Cronbach’s alpha for most of them, which suggests such items contributed to the internal consistency of this dimension (Table 5). However, removing one of the items in “intrinsic value” subdimension (“I often question the meaning of household and family work, since I have to start all over again every day”) raised the Cronbach’s alpha value from 0.45 to 0.57. Besides that, removing one item from overcommitment dimension (“If I postpone something that I really should have finished today, I have trouble sleeping at night”) raised the internal consistency value of the subdimension from 0.80 to 0.83.

**Table 5:** Cronbach’s alpha coefficient of the scores in the dimensions of the effort-reward imbalance scale in household and family work in case an item were removed. Test-retest reliability study Rio de Janeiro, RJ, Southeastern Brazil, 2013. (N = 1,045)

Domestic ERI	Item	Cronbach’s alpha of the dimension	Cronbach’s alpha of the dimension and removed item
Overcommitment	1. From the moment I wake up in the morning, I often begin to worry about household and family work that needs to be completed.	0.80	0.76
	2. I constantly think about my responsibilities at home, and I’m still preoccupied with them in the evening.		0.70
	3. I easily run into time pressures in my household and family work.		0.72
	4. If I postpone something that I really should have finished today, I have trouble sleeping at night.		0.83
Effort	1. Frequently there is great time pressure due to the many tasks in household and for my family.	0.93	0.92
	2. I am frequently interrupted and disturbed in my activities in the household and for my family.		0.93
	3. Often I feel as never being off duty.		0.92
	4. I would need more hours in the day in order to accomplish all my household and family work.		0.92
	5. Over the last years, my household and family work have become more extensive.		0.92
	6. In household and family work, I often have the feeling of having to accomplish ‘a thousand things’ all at the same time.		0.91
	7. I often feel overwhelmed by the large number of household and family responsibilities.		0.92
	8. I hardly get a moment’s rest during the day because of the many demands placed on me by the household and my family		0.93
Reward	Intrinsic value 1. I feel that overall, household and family work are worth the effort.	0.45	0.24
	2. I often question the meaning of household and family work, since I have to start all over again every day.		0.57
	3. The work I do for my family provides a deeper meaning to my life.		0.25
	Societal esteem 4. In my interactions with other people, I often have the experience that the roles of housewife and mother are poorly recognized and appreciated.	0.40	0.22
	5. Nowadays, a person is regarded disapprovingly if he/she is ‘only’ involved in household and family work.		0.33
	6. The fact that household and family work are unpaid seems unjust to me.		0.37
	Recognition from the partner 7. I usually obtain an appropriate level of recognition and appreciation from my partner for my work at home.	0.84	0.74
	8. Often my partner does not notice my work in the household and for the family.		0.80
	9. My partner often thanks me for my work at home.		0.80
	Affection from the children* 10. From my child/children I usually feel the appreciation and affection that I would wish for.	0.72	-
	11. I receive a great deal in return from my children/child for my efforts at home.		-

* Cronbach’s alpha in case the removed item cannot be calculated as a function of the number of items in the subdimension.

## DISCUSSION

The results in this study showed that the Brazilian version of domestic ERI was found to fall within an acceptable range regarding the time stability of its items, which were evaluated using the test-retest reliability analysis. Besides that, they suggest that most items be adjusted in their respective dimensions by having their internal consistency evaluated. Each step in the process of transculturally adapting the scale to the Brazilian culture, including the initial psychometric evaluation, was conducted according to the criteria from the specialized literature^6^. The changes required in the scale were based on the discussions between the researchers in charge and specialists, as well as on advice from one of the authors of the original scale, Stephanie Sperlich.

The education levels of the subjects allowed them to fill out the questionnaires regarding the domestic ERI scale by themselves in the test-retest reliability analysis, which prevented sources of variability between interviewers from arising. No missing information was observed in the questionnaires, which suggested that the items in the scale were clear and well understood.

The test-retest reliability analysis of the items and of the dimensions in the scale for evaluating effort-reward imbalances in household and family work was found to have proper levels and good questionnaire stability in the different dimensions, according to the predefined criteria. For most items, time stability as evaluated by the kappa index was partially found to be related to the high frequencies of positive answers in our population, as they were found to be high after being adjusted for prevalence and bias. We should also point out that the items with the lowest time stability values are also included in subdimensions with lower Cronbach’s alpha values (reward and societal esteem). However, we did not find any other studies analyzing the test-retest reliability of the scale, which prevents it from being compared to others.

As in the original study^17^, the results found have satisfactory internal consistency for most dimensions in the domestic ERI scale, with values that are very similar in dimensions overcommitment (alpha = 0.81), effort (alpha = 0.92), and recognition from the partner (alpha = 9.82). Besides that, the German study authors also found lower internal consistency values in subdimensions intrinsic value and societal esteem, and these were yet higher than the ones found in this article (respectively, alpha = 0.69 and alpha = 0.73). Nonetheless, some differences between this investigation and the German study limit comparing the results between each other, as the latter was developed with a wide range of professions and included women who were exclusively dedicated to household duties. It is also possible that cultural differences regarding the recognition of intrinsic value and societal esteem may have different meanings in both contexts. However, we suggest the performance of qualitative studies to allow us to capture the meaning of the items in these dimensions among Brazilian workers, and to understand them. Important elements such as age, number of children, exclusive dedication to household and family work, and partner’s participation in these tasks must be considered in future investigations that make use of the domestic ERI scale, because of its relationship with the domestic workloads of women.

Although this study comprised a sample of female nursing workers with different characteristics (nighttime and daytime workers with different education levels), including a restricted category limits the generalization of results to the general population of female workers. The process shown was fundamental for the inclusion of a questionnaire in a new context; however, the steps conducted do not necessary mean the relevance of the study was fully examined. There are complementary psychometric evaluations of the questionnaire in the context of the studied population that are still in progress. In this phase, construct validity will be examined, including the dimensional structure, the relevance of the items in the respective dimensions and subdimensions, and construct validity.

Some authors^7^ ^,^ ^11^ criticize the use of Cronbach’s alpha as the only tool to estimate the internal consistency of a questionnaire. However, a recent study^16^ pointed this indicator out as a more conservative one; i.e., it yields values that are inferior to the ones of other estimators, such as McDonald’s Omega. Besides that, using Cronbach’s alpha allowed direct comparison to the original study. Finally, the debate on to which extent household and family work contributes to health problems in female populations has been highlighted in the literature^1^. Domestic ERI scale allows investigating aspects that involve women’s illnesses and health, which are related to stress in a scenario that is so specific of the female universe. However, the questions involving participation in domestic activities, in the work-health relationship, are not restricted to accumulating duties. Therefore, the transcultural adaptation of this questionnaire to Brazilian Portuguese, whose steps are shown in this text, may help composing an initial panorama of household and family work in Brazil. The results indicate proper time stability for the items in the scale, which suggests it can be properly used in populations whose characteristics are similar to the ones in this study. Applying the questionnaire in other categories would reinforce the process and favor the evaluation of whether the questionnaire is relevant for the general population of female workers. Besides that, the scale may be useful in the evaluation of psychosocial stress, considering the professional and domestic realms, thus opening new perspectives for analyzing female work in all its dimensions and meanings.
